# Yolk sac tumor in a patient with transverse testicular ectopia

**DOI:** 10.1186/1477-7819-9-91

**Published:** 2011-08-16

**Authors:** Yi-Ping Zhu, Shi-Lin Zhang, Ding-Wei Ye, Guo-hai Shi, Wen-jun Xiao

**Affiliations:** 1 Department of Urology, Fudan University Shanghai Cancer Center, China; 2 Department of Oncology, Shanghai Medical College, Fudan University, Shanghai, China

## Abstract

Transverse testicular ectopia (TTE) is a rare anomaly in which both testes descend through a single inguinal canal. We report a case of yolk sac tumor in the ectopic testis of a patient with TTE. A 24-year-old man presented to our hospital with a left inguinal-mass, right cryptorchidism and elevated alpha-fetoprotein (AFP). A left herniotomy 3 years earlier demonstrated both testes in the left scrotum, one above another positionally. Four months ago, a left scrotal mass appeared and radical orchiectomy of both testes revealed testicular yolk sac tumor of the ectopic testis. An enlarging left inguinal-mass appeared 2 months ago and he was referred to our hospital. Laboratory data showed an elevation of AFP (245.5 ng/ml) and a 46 XY karyotype. He underwent bilateral retroperitoneal lymph node dissection and simultaneous left inguinal mass dissection. Histopathologic examination revealed a diagnosis of recurrent yolk sac tumor in the left inguinal mass. The retroperitoneal lymph node was not enlarged and, on histopathology, was not involved. The patient has now been followed up for 8 months without evidence of biochemical or radiological recurrence.

## Background

Transverse testicular ectopia (TTE), also named crossed testicular ectopia, is a rare anomaly in which both testes descend through a single inguinal canal while the opposite inguinal canal and hemiscrotum are empty. More than 100 cases of TTE have been reported since Von Cenhossek described the first case in 1886[1-3]. As with undescended testis, the ectopic gonads are at increased risk of malignant transformation [4]. We report a case of yolk sac tumor in the ectopic testis of a patient with TTE.

## Case Presentation

**Figure 1:**
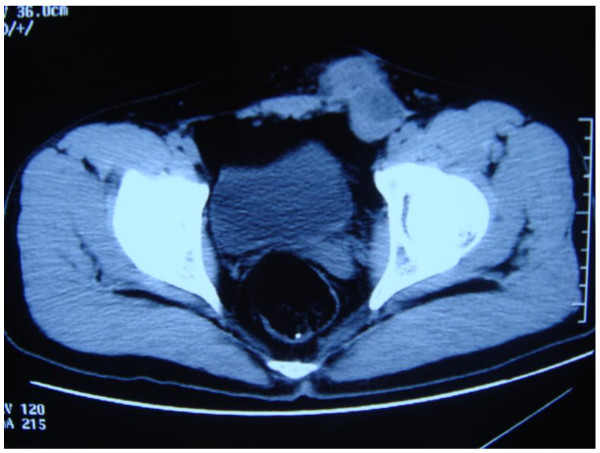
**Left inguinal mass on the CT scan**.

A 24-year-old man, who had fathered a child, presented to our hospital with a left inguinal-mass (Figure 1), right cryptorchidism and elevated alpha-fetoprotein (AFP) (245.5 ng/ml, normal 0.6 to 6.7 ng/ml). He had no family history of persistent Mullerian duct syndrome or testicular tumors. His past surgical history included a left herniotomy 3 years earlier during which both testes were demonstrable in the left scrotum, with one located above the other positionally. Four months ago, a left scrotal mass appeared and elevated AFP (373.5 ng/ml) was detected. Carcinoembryonic antigen (CEA) and human chorionic gonadotropin (HCG) levels were within normal ranges. Radiological staging prior to management of the scrotal mass revealed no metastasis. During surgery, the normal-position testis was found to be invaded by the ectopic malignant one. Radical orchiectomy of both testes revealed a yolk sac tumor in the ectopic one. The AFP level was still high (64.7 ng/ml) after orchiectomy. An enlarging left inguinal mass appeared 2 months ago and he was referred to our hospital.

On admission, physical examination revealed a normal constitution, with secondary sex characteristics and normal external genital development. His karyotype was 46 XY. Laboratory data showed an elevation of AFP (245.5 ng/ml). CEA and HCG levels, however, were within normal ranges. Chest x-ray and abdominal CT were normal.

**Figure 2:**
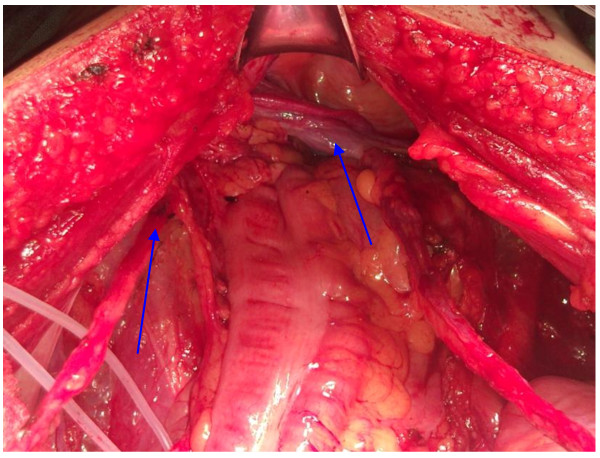
**Both spermatic cords were found to descend through the left internal inguinal ring**.

**Figure 3:**
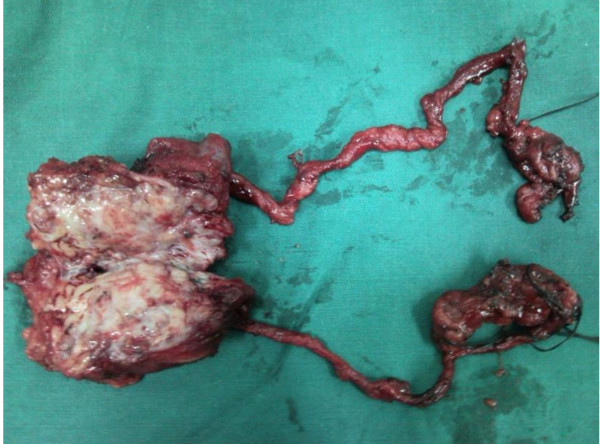
**The resected left inguinal mass with bilateral spermatic cords and retroperitoneal lymph nodes**.

The patient underwent open bilateral modified retroperitoneal lymph node dissection (RPLND) and simultaneous left inguinal mass dissection at our hospital. During operation, bilateral spermatic cords were found to descend through the left internal inguinal ring (Figure 2) and no persistent Mullerian duct structures were observed. Histopathologic examination revealed a diagnosis of yolk sac tumor in the recurrent left inguinal mass at the spermatic residual end (Figure 3). The retroperitoneal lymphadenopathy was negative for metastasis. The plasma levels of AFP had returned to normal at the 3-month postsurgical visit. He was followed for 8 months without evidence of biochemical or radiological recurrence.

## Discussion

TTE is a rare form of testicular ectopia. The clinical presentation generally includes an inguinal hernia on one side and a contralateral or sometimes bilateral cryptorchidism [5]. In the majority of published reports, the exact diagnosis was established only during surgical intervention [6]. Recently, it has been suggested that magnetic resonance imaging (MRI) would be useful for preoperative localization of impalpable testis[7]. Laparoscopy is useful for both the diagnosis and management of TTE and its associated anomalies[8].

TTE has been classified into three types on the basis of associated anomalies: (1) associated with inguinal hernia alone (40% to 50%); (2) associated with persistent or rudimentary müllerian duct structures (30%); and (3) associated with other anomalies without müllerian remnants (inguinal hernia, hypospadias, pseudohermaphroditism, and scrotal abnormalities) (20%)[2]. According to this classification, our patient would be designated as a case of type 1 TTE.

Patients with TTE are at increased risk of malignant transformation, with a 5%-18% overall incidence rate, similar to the rate of cryptorchidism[4]. There have been reports of embryonal carcinoma, seminoma, yolk sac tumor, teratoma and mixed germ cell tumors [9-11]. However, most reported cases of intraabdominal tumors were in patients with type 2 TTE. To the best of our knowledge, we are reporting the first case of a scrotal yolk sac tumor in a patient with type 1 TTE.

Therapeutic approaches for TTE include transseptal orchiopexy or extraperitoneal transposition of the testis and a search for müllerian remnants and other anomalies[2]. However, the malignant potential of these gonads must be recognized and orchiopexy, even if performed early in life, does not decrease the risk of malignancy[11]. As most tumors in this group will occur after puberty, long-term follow-up is mandatory. Some surgeons suggest that orchiectomy should be performed in patients older than 2 years because orchiopexy offers only limited protection against future malignancy[11]. In our opinion, for patients who have not yet reached puberty, orchiectomy is indicated only for testis that cannot be mobilized to a palpable location. For adult patients, dissection of the ectopic testis and active surveillance of the remnant is recommended.

This patient is classified as having a clinical stage I nonseminomatous germ cell tumor (NSGCT) of the ectopic testis. Histopathologic examination revealed a diagnosis of yolk sac tumor in the recurrent left inguinal mass. Such patients can be managed by surveillance, primary chemotherapy or nerve-sparing RPLND.. Surveillance can spare patients without metastasis from undergoing chemotherapy or RPLND. However, according to Foster's review, approximately 30% of patients with clinical stage I NSGCT have occult metastases that are difficult to identify with imaging[12]. In addition, prepubertal testicular neoplasms differ greatly from postpubertal lesions. Most prepubertal patients (85%) with yolk sac tumor are stage I at presentation, compared with only 35% stage I tumors at presentation of postpubertal patients[13]. Therefore, nerve-sparing RPLND or platinum-based chemotherapy has a central role in management of postpubertal adult patients.

Although platinum-based chemotherapy for yolk sac tumors has produced excellent survival results, its side effects are not negligible. Raynoud's phenomenon (25-30%), ototoxicity (20%), neurotoxicity (15%), and nephrotoxicity (31%) are some of the common complications that occur in survivors of testicular cancer [14]. The advantages of RPLND include the immediate determination of exact stage, the chance for cure with surgical removal of involved lymph nodes and the elimination of the need for monitoring a patient postoperatively with CT scans. Although the surgery itself is a burden to most patients, we treated our patient with bilateral modified RPLND along with excision of the inguinal mass after discussions with the patient and his wife. No further chemotherapy was employed because retroperitoneal lymph nodes showed no metastases.

## Conclusion

We present a rare case of a scrotal yolk sac tumor in a patient with type 1 TTE. The diagnosis of TTE should be considered when unilateral hernia and concurrent cryptorchidism of the contralateral side are present. The malignant potential of the ectopic gonads must be recognized and long-term follow-up is mandatory. For patients who have not yet reached puberty, orchiopexy is recommended and orchiectomy is indicated only for testes that cannot be mobilized to a palpable location. For adult patients, dissection of the ectopic testis and active surveillance of the remnant is recommended. For patients with scrotal yolk sac tumor and type 1 TTE, RPLND, surveillance and adjuvant chemotherapy are all accepted treatments for long-term survival after orchiectomy.

## Consent

Written informed consent was obtained from the patient for publication of this case report and any accompanying images. A copy of the written consent is available for review by the Editor in Chief of this journal.

## Competing interests

The authors declare that they have no competing interests.

## Authors' contributions

YPZ, SLZ and DWY conceived the concept, participated in drafting the manuscript, and conducted critical review. GHS and WJX took part in the care of the patient, assembled data, and participated in writing the manuscript. All authors read and approved the final manuscript.
